# In-depth proteome characterization of endometrium and extraembryonic membranes during implantation in pig

**DOI:** 10.1186/s40104-024-01002-x

**Published:** 2024-03-12

**Authors:** Maria A. Gil, Josep M. Cambra, Heriberto Rodriguez-Martinez, Cristina Cuello, Inmaculada Parrilla, Emilio A. Martinez

**Affiliations:** 1 Department of Medicine and Animal Surgery, International Excellence Campus for Higher Education and Research “Campus Mare Nostrum”, University of Murcia, Murcia, Spain; 2 Institute for Biomedical Research of Murcia (IMIB-Pascual Parrilla), Murcia, Spain; 3 Technical University of Munich, Munich, Germany; 4 Department of Biomedical & Clinical Sciences (BKV), BKH/Obstetrics & Gynaecology, Linköping University, Linköping, Sweden

**Keywords:** Endometrium, Extraembryonic membranes, Implantation, Pig, Proteome

## Abstract

**Background:**

Proteome characterization of the porcine endometrium and extraembryonic membranes is important to understand mother-embryo cross-communication. In this study, the proteome of the endometrium and chorioallantoic membrane was characterized in pregnant sows (PS) during early gestation (d 18 and 24 of gestation) and in the endometrium of non-pregnant sows (NPS) during the same days using LC-MS/MS analysis. The UniProtKB database and ClueGO were used to obtain functional Gene Ontology annotations and biological and functional networks, respectively.

**Results:**

Our analysis yielded 3,254 and 3,457 proteins identified in the endometrium of PS and NPS, respectively; of these, 1,753 being common while 1,501 and 1,704 were exclusive to PS and NPS, respectively. In addition, we identified 3,968 proteins in the extraembryonic membranes of PS. Further analyses of function revealed some proteins had relevance for the immune system process and biological adhesion in endometrium while the embryonic chorion displayed abundance of proteins related to cell adhesion and cytoskeletal organization, suggesting they dominated the moment of endometrial remodeling, implantation and adhesion of the lining epithelia. Data are available via ProteomeXchange with identifier PXD042565.

**Conclusion:**

This is the first in-depth proteomic characterization of the endometrium and extraembryonic membranes during weeks 3 to 4 of gestation; data that contribute to the molecular understanding of the dynamic environment during this critical period, associated with the majority of pregnancy losses.

**Supplementary Information:**

The online version contains supplementary material available at 10.1186/s40104-024-01002-x.

## Background

The female reproductive tract provides a unique environment for a successful pregnancy. It is imperative that the non-pregnant uterus be transformed into a capable environment for the establishment and maintenance of pregnancy. To achieve this, intimate cross-communication between the endometrium and the embryo is necessary at an early stage of life [1], and this dialog can influence subsequent fetal developmental potential [2] and even post-natal performance. Initially, the trophoblast-derived estrogen is one of the most important embryonic signals to activate the maternal uterus for attachment [3, 4]. Once the conceptus has established its position in the uterus (> d 15), its development and growth also require many other maternal-embryonic cellular and molecular interactions to ensure substantial vascular changes in the endometrium and the chorioallantois, mainly development of capillaries under the lamina propriae, essential to provide full function to the pig epitheliochorial placenta required for a successful pregnancy. It is during the peri-attachment window between d 12–30 of gestation when most (20%–30%) of the embryos produced in pig natural or artificial breeding die [5]; embryonic death with a substantial impact on pig production efficiency, especially because it significantly limits litter size.

Different studies have focused on the elucidation of the complex embryo-maternal communication network to reduce pregnancy loss. Among these, several studies examined gene expression during the peri-implantation stage, when the majority of embryonic losses occur, or have compared the transcriptomic profiles of pregnant and non-pregnant animals. Interestingly, these studies identified changes in the expression of genes that can directly or indirectly contribute to reproductive success such as genes related to cell proliferation, hormone synthesis and metabolism, cell adhesion or those related to cytokine production and immune local response [1, 6–10]. However, we should not forget that changes in gene expression do not always lead to a corresponding alteration in the expression of proteins, which are crucial components in all biological processes. The knowledge of the proteome is therefore equally relevant to gene expression changes, being able to detect both normal and abnormal physiological conditions. Proteome characterization can lead to a better understanding of physiological processes and to identify proteins that may serve as potential biomarkers. To date, only a limited number of studies have unfortunately explored the protein expression profiles of the pig endometrium during the crucial period of maternal recognition of pregnancy, which occurs between d 9 and 13 [11, 12] or during mid/last-gestation, from 40 to 93 days of pregnancy [13, 14]. These studies have identified several proteins associated with endometrial function, which could play a role in maternal recognition and progression of pregnancy. Therefore, in order to fully understand the molecular interaction between the conceptus and its chorioallantois with the endometrium, we have aimed to characterize the proteome profile of both, the non-pregnant (control) endometrium compared with that of the “pregnant” endometrium and also characterized the proteome profile of the extraembryonic membranes between the 3^rd^ and the 4^th^ week period, when the major conceptus loss occurs. Our research hypothesis focused on the identification of proteins associated with specific functions related to the immune system process and biological adhesion that may contribute to the molecular understanding of mother-embryo cross-communication during implantation.

## Material and methods

### Animals

All experiments were conducted in compliance with international guidelines (Directive 2010/63/EU) and were approved by the Bioethical Committee for Animal Experimentation at the University of Murcia, Spain (research code: 522/2019; 2019/03/21). For this study, Landrace × Large-White sows, parity 2 to 7 (aged 1.5–3.5 years) with weight around 260–300 kg, were randomly selected from a commercial farm (Agropor SA, Murcia, Spain) immediately after weaning.

Mature boars (aged 2–3 years) with a proven record of fertility were selected as semen donors from a commercial breeding boar station (AIM Iberica, Spain). All animals were provided with ad libitum access to water and fed with commercial diets that met their nutrient requirements.

### Estrus detection

Estrus detection was conducted as described previously [15] by snout-to-snout contact between females and a mature boar and applying back-pressure twice daily, starting one day after weaning. Only sows showing signs of estrus on d 4 after weaning were used for the experiment.

### Experimental design

To characterize the proteomic profile of porcine endometrium and extra-embryonic membranes during early pregnancy, sows were post-cervically inseminated at 6 and 24 h after the onset of estrus (d 0 = onset of estrus) with 40 mL doses containing 1.5 × 10^9^ live (for pregnant sows; PS) or dead spermatozoa (for non-pregnant sows; NPS, controls). The sows were euthanised on d 18 and 24 after insemination and their uteri were opened lengthwise along the anti-mesometrial side. In PS, pregnancy was verified by recovering conceptuses from both uterine horns, and both endometrial and extra-embryonic membranes samples were carefully cut out and pooled from 3 different areas of attachment in d 18 of gestation (*n* = 4 sows) or 3 different implantation areas in d 24 of gestation (*n* = 4 sows). In NPS, morphological examination of the ovaries immediately after euthanasia confirmed the presence of active corpora lutea (between 16–23 per sow) and endometrial tissues were cut out and pooled from 3 different areas at random of the uterine horn on d 18 (*n* = 4 sows) or d 24 (*n* = 4 sows). All samples were collected by the same technician, immersion-frozen in liquid nitrogen and preserved frozen at −80 ºC until analysis.

### Proteomics analysis

Proteome analyses were conducted at the Proteomics Unit of the University of Valencia, which is affiliated with ProteoRed, PRB2-ISCIII, located in Valencia, Spain.

#### Protein extraction

For extraction of proteins from the endometrium or membranes, a 2D Grinding kit (GE Healthcare Life Sciences, United Kingdom) was used with 150 μL of lysis buffer [7 mol/L Urea, 2 mol/L thiourea, 4% 3-[(3-cholamidopropyl) dimethylammonio]-1-propanesulfonate (CHAPS; Sigma-Aldrich, Madrid, Spain), and 30 mmol/L Tris (pH 8.5)]. The quantity of protein in each sample was determined through analysis with an RC-DC kit (Bio-Rad, Richmond, CA, USA).

#### Spectral library building

For generation of a spectral library from one-dimensional sodium dodecyl sulfate-polyacrylamide gel electrophoresis (1D SDS-PAGE; Bio-Rad, Richmond, CA, USA), aliquots containing the same amount of each sample type were combined to form a single pool. Endometrial samples from PS and NPS were divided into two different libraries with extra-embryonic samples kept in a separate library.

##### Gel protein digestion

Each library’s respective career was divided into 8 pieces and then subjected to digestion using Sequencing Grade Trypsin (Promega Corporation, Madison, WI, USA) following the method described by Shevchenko et al. [16]. After trypsin digestion, 10% trifluoroacetic acid (TFA; Fisher Scientific, Madrid, Spain) was added to stop the reaction, and the resulting supernatant was removed. The sliced library gels were then dehydrated using pure acetonitrile (ACN; Fisher Scientific, Madrid, Spain). The resulting peptide solutions were combined with their corresponding supernatants, and the resulting peptide mixtures were dried in a speed vacuum. Prior to LC-MS/MS analysis, the dried peptides were resuspended in a solution containing 2% ACN and 0.1% TFA.

##### LC-MS/MS analysis

For the analysis, 5 μL of each digested pool was loaded onto a trap column (NanoLC Column, 3 μm C18‐CL, 75 μm × 15 mm; Eksigent Technologies, Dublin, CA, USA) and desalted with 0.1% TFA at a flow rate of 3 μL/min for 5 min. Next, the peptides were loaded onto an analytical column (LC Column, 3 μm C18‐CL, 75 μm × 12 cm, Nikkyo Technologies, Tokyo, Japan) that had been equilibrated with a solution containing 5% ACN 0.1% formic acid (FA; Fisher Scientific, Madrid, Spain). Peptide elution was carried out using a linear gradient of 5% to 40% of solvent B (0.1% FA in ACN) over a period of 120 min, with a flow rate of 300 nL/min. Finally, the eluted peptides were analyzed using a mass spectrometer nanoESIqQTOF (5600 TripleTOF, AB SCIEX, Framingham, MA, USA).

The TripleTOF was set to information‐dependent acquisition mode. This involved conducting a 250‐ms time of flight (TOF) MS scan within the range of 350–1,250 *m/z*, followed by 150‐ms product ion scans within the range of 350–1,500 *m/z* on the 25 most intense ions with charges ranging from 2 to 5. To ensure consistency, the rolling collision energy equations were applied to all ions with a charge of 2+. These equations were determined by the following parameters: |CE|= (slope) × (*m/z*) + (intercept), where for 2+ ions, the slope was 0.0625 and the intercept was −3.

##### Protein identification

The peak list was generated using the ProteinPilot search engine (version 5.0 SCIEX) directly from the 5600 TripleTof wiff files. The Paragon algorithm of proteinPilot [17] was employed for searching the UniProt database with specific parameters, which included trypsin specificity, cys-alkylation, taxonomy mammalian restricted, and thorough search effort. FDR correction was also applied to ensure accurate protein identification. To prevent the use of identical spectral evidence in multiple proteins, the Pro Group algorithm was used to group the identified proteins. The Pro Group Report defined a protein group as a collection of proteins that share some physical evidence. Pro Group differs from sequence alignment analyses in that it relies solely on observed peptides for the formation of protein groups, rather than comparing complete theoretical sequences. As the observed peptides are derived from experimentally obtained spectra, the formation of protein groups in Pro Group can be viewed as being guided by the utilization of spectra. Consequently, unobserved regions of the protein sequence are irrelevant in elucidating the data.

The mass spectrometry proteomics data have been deposited to the ProteomeXchange Consortium via the PRIDE [18] partner repository with the dataset identifier PXD042565.

#### Data analysis

The UniProtKB database (www.uniprot.org) was used to conduct bioinformatic analysis of the identified endometrium and extraembryonic proteins. This analysis was aimed to acquire functional Gene Ontology (GO) annotation, specifically in the categories of “molecular function”, “cellular component”, and “biological processes”.

Venn diagrams of the endometrial proteins were created to identify common and unique proteins of PS and NPS using Venny online software (https://csbg.cnb.csic.es/BioinfoGP/venny.html).

Cytoscape 3.9.1 software and ClueGO v2.5.9 were utilized to generate an interaction network of unique proteins involved in immune system process, biological adhesion and reproductive process. The following selection criteria were applied: (1) a *P*-value cut-off of at least 0.05, (2) use of the enrichment/depletion statistical test (two-sided hypergeometric test) with Bonferroni step down, minimum GO level 2, maximum GO level 6, and a kappa score threshold of 0.4, and (3) analysis of Homo sapiens organism with genes present in KEGG pathways, REACTOME pathways and GO Biological Process.

### Western blotting

In order to validate the LC-MS/MS results, total protein was extracted from the three pools of samples (endometrial samples from PS and NPS and extra-embryonic samples) to perform Western blot analysis. Briefly, 100 mg of frozen tissues were disrupted in a polytron homogenizer using RIPA lysis buffer with protease and phosphatase inhibitor cocktails and quantified with the Bradford assay using BSA as standard (Protein Assay Kit, Bio-Rad, Hercules, CA, USA). Total protein extracts (30 mg) were mixed with 5 × SDS sample buffer (62.5 mmol/L Tris-HCl, pH 6.8, 2% SDS, 10% glycerol, 5% b-mercaptoethanol, and 0.005% bromophenol blue) and resolved by SDS-PAGE on 10% acrylamide gels. Proteins were detected immunologically following electrotransfer onto 0.45-µm pore–size nitrocellulose membrane (Bio-Rad). The membranes were blocked with 5% BSA in TBS and 0.1% Tween-20 for 1 h at room temperature and incubated for 1 h with continuous agitation with primary antibodies. The antibodies used in this study were: anti-heme oxygenase 1 (rabbit polyclonal, 1:2,000 dilution, Abcam, Cambridge, UK), anti-RPS6 (rabbit polyclonal, 1:5,000 dilution, Abcam), anti-PTN (goat polyclonal, 1:2,000 dilution, Abcam), and anti-thrombospondin-1 (rabbit polyclonal, 1:5,000 dilution, Abcam). Blots were washed three times for 10 min in TBS and 0.1% Tween-20 and incubated with horseradish peroxidase-conjugated (HRP) secondary antibodies for 1 h at room temperature with continuous agitation: HPR-conjugated goat anti-rabbit IgG antibody (1:5,000 dilution, Merck KGaA, Darmstadt, Germany) and HPR-conjugated rabbit anti-goat IgG antibody (1:5,000 dilution, Merck KGaA). Blots were developed using a peroxidase reaction with the enhanced chemiluminescent immunoblotting detection system (ECL-Plus, GE Healthcare, Little-Chalfont, Buckinghamshire, UK). Antibodies were accepted when they displayed a single predominant band at the expected molecular weight.

## Results

### Analysis of the endometrial proteome profile

Using FDR correction, the UniProt mammalian library enabled the identification of 3,254 proteins in the endometrium of PS during the peri-implantation period (d 18 and 24 of gestation). Similarly, 3,457 proteins were identified in the endometrium of NPS on the same days of the cycle. Additional file 1: Tables S1 and S2 contain a comprehensive list of all proteins identified, along with their corresponding unused score, percentage of sequence coverage, UniProt accession number, protein name, species, and matched peptides. Both endometrial proteomes featured high-abundance proteins (Tables 1 and 2) that were primarily associated with ion transport and binding. Examples include albumin, hemoglobin subunit beta, and globin domain-containing protein. Both endometrial proteomes also contained proteins related to cytoskeleton organization, such as myosin-9 and plectin, while filamin A was exclusive to the endometrial proteome of PS. Proteins involved in inflammatory and immune responses of the endometrium, such as complement C3 and IgG heavy chain proteins, were also present in both PS and NPS proteomes, while the proteome of NPS also included Ig-like domain-containing protein.

**Table 1:** Summary of high abundance proteins in the endometrium of pregnant sows (PS) during implantation

Protein name	Gene symbol	UniProt ID	Molecular function	Biological process	Matched peptides
Albumin	*ALB*	F1RUN2_PIG	Enables metal ion binding		735
Hemoglobin subunit beta	*HBB*	F1RII7_PIG	Enables oxygen transport and binding	Involved in oxygen transport, cellular oxidant detoxification	481
GLOBIN domain-containing protein	*LOC110259958*	F1RGX4_PIG	Enables oxygen transport and binding	Involved in oxygen transport, cellular oxidant detoxification	384
Beta-1 metal-binding globulin	*TF*	B3CL06_PIG	Enables metal ion binding	Involved in ion transport	378
Alpha-2-macroglobulin	*A2M*	K9J6H8_PIG	Enables peptidase inhibitor activity	Involved in negative regulation of endopeptidase activity	319
Complement C3	*C3*	F1SBS4_PIG	Enables endopeptidase inhibitor activity	Involved in complement activation, inflammatory response, immune system process	293
IgG heavy chain	*IGHG*	L8B0R9_PIG		Involved in immune system process	289
Myosin-9	*MYH9*	K9IVP5_PIG	Enables cytoskeletal motor activity, ATP binding, actin filament binding	Involved in phagocytosis	239
Filamin A	*FLNA*	A0A286ZXU2_PIG	Enables actin filament binding	Involved in actin cytoskeleton organization	233
Plectin	*PLEC*	A0A287B9H1_PIG	Enables structural constituent of cytoskeleton and structural molecule activity and actin binding	Involved in intermediate filament cytoskeleton organization, in wound healing and in leukocyte migration in immune response	221

**Table 2:** Summary of high abundance proteins in the endometrium of non-pregnant sows (NPS) at d 18 and 24 of the cycle

Protein name	Gene symbol	UniProt ID	Molecular function	Biological process	Matched peptides
Albumin	*ALB*	F1RUN2_PIG	Enables metal ion binding		641
Serotransferrin	*TF*	TRFE_PIG	Enables metal ion binding	Involved in ion transport and homeostasis	406
Hemoglobin subunit beta	*HBB*	F1RII7_PIG	Enables oxygen transport and binding	Involved in oxygen transport, cellular oxidant detoxification	367
Plectin	*PLEC*	A0A287BNK7_PIG	Enables cytoskeleton protein binding and actin binding	Involved in intermediate filament cytoskeleton organization	308
Alpha-2-macroglobulin	*A2M*	K9J6H8_PIG	Enables peptidase inhibitor activity	Involved in negative regulation of endopeptidase activity	301
Complement C3	*C3*	F1SBS4_PIG	Enables endopeptidase inhibitor activity	Involved in complement activation, inflammatory response, immune system process	285
GLOBIN domain-containing protein	*LOC110259958*	F1RGX4_PIG	Enables Oxygen transport and binding	Involved in Oxygen transport, cellular oxidant detoxification	245
Ig-like domain-containing protein	*N/A*	F1STC5_PIG	Enables immunoglobulin receptor binding and antigen binding	Involved in immune system process, complement activation, phagocytosis, engulfment, recognition	243
IgG heavy chain	*IGHG*	L8B180_PIG		Involved in immune system process	233
Myosin-9	*MYH9*	K9IVP5_PIG	Enables cytoskeletal motor activity, ATP binding, actin filament binding	Involved in phagocytosis	223

#### Gene Ontology analysis of the endometrial proteome

**Fig. 1:**
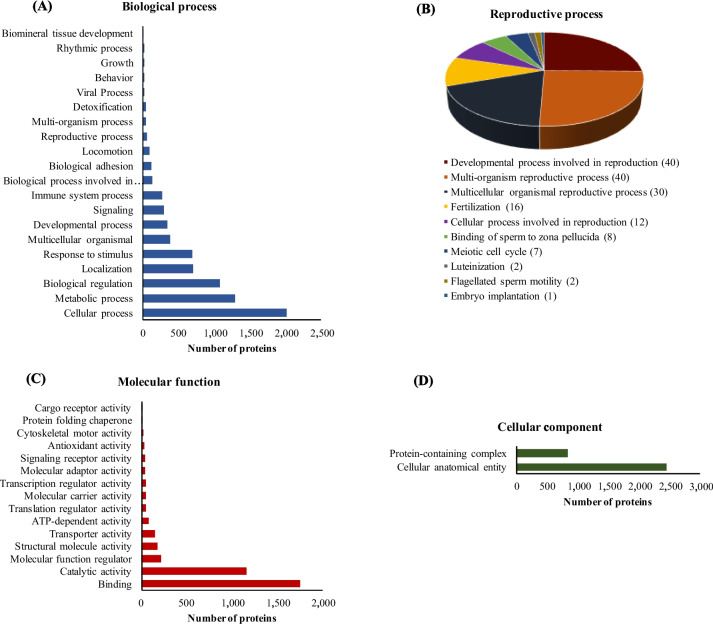
Gene Ontology analysis of the endometrial proteome of pregnant sows during implantation. **A** Biological process. **B** Reproductive process. **C** Molecular function. **D** Cellular component

Over the 3,254 proteins identified in the UniProt mammalian endometrial library of PS, a total of 3,122 proteins were successfully mapped to UniProtKB IDs, from which 2,484 proteins had biological process annotation information (Fig. 1A). The highest proportion included cellular and metabolic processes and biological regulation. However, there were also 349, 268, 115 and 62 proteins involved in developmental process, immune system process, biological adhesion, and reproductive process, respectively. Most of the proteins with reproductive process function were related to developmental processes associated with reproduction and multi-organism and multi-cellular organismal reproductive processes (Fig. 1B). There were also 16 and 12 proteins enclosed to fertilization and cellular process involved in reproduction, respectively and two proteins, alpha-2-macroglobulin and platelet-derived growth factor receptor alpha related to luteinization term. When the proteins with information on molecular function were analyzed, most of them were enclosed into binding and catalytic activity (2,408 proteins; Fig. 1C). Out of the 2,474 proteins with cellular components information, most of them were associated with the cellular anatomical entity (Fig. 1D).

**Fig. 2:**
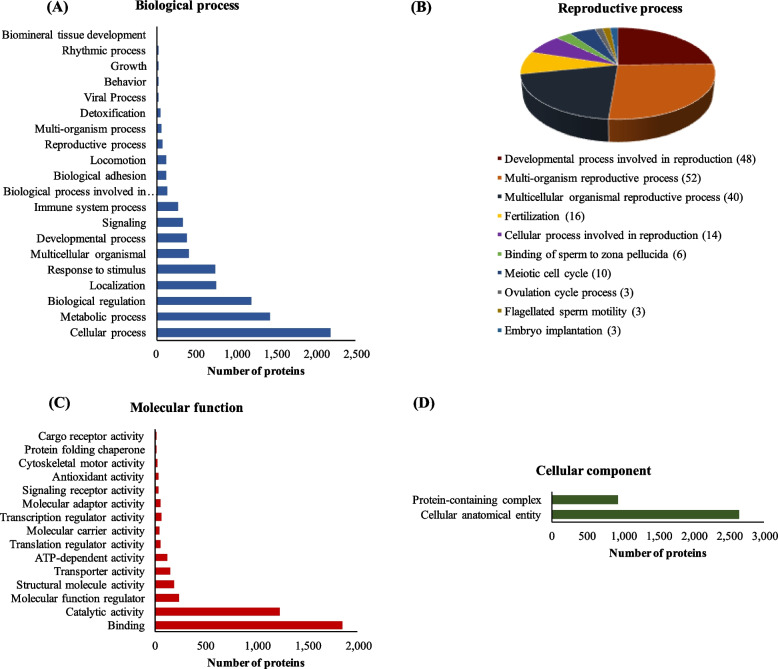
Gene Ontology analysis of the endometrial proteome of non-pregnant sows at d 18 and 24 of the cycle. **A** Biological process. **B** Reproductive process. **C** Molecular function. **D** Cellular component

A similar GO functional classification was evident in the endometrium of NPS. Of the 3,457 proteins identified in the UniProt mammalian library, a total of 3,348 proteins were successfully mapped to UniProtKB IDs. Among these, 2,658, 2,547 and 2,685 proteins were annotated with information related to biological process, molecular function and cellular component, respectively (Fig. 2). Out of the 68 proteins that were found to contain information regarding reproductive process in the NPS (Fig. 2B), 10 proteins had meiotic cell cycle annotation, while 3 proteins were related to the ovulation cycle process (myosin-9, progesterone receptor and platelet-derived growth factor receptor alpha).

We compared the two endometrial proteomes to check identified proteins that were unique to either the PS or the NPS proteome, as well as those that were common to both. As a result, we found that 1,753 proteins were detected in both groups of samples, while 1,501 proteins were exclusively detected in PS samples, and 1,704 proteins were solely identified in NPS samples. The lists of proteins identified in both groups and found only in PS and NPS samples are available in Additional file 1 (Tables S3–S5). In this context, and to clarify the proteomic profile of both groups, we perform the categorization of the unique proteins according to the protein class ontology. Upon conducting a functional GO analysis of the unique proteins present in the PS endometrial samples using the UniProtKB database, it was observed that 53 and 23 proteins were involved in immune system process and biological adhesion, respectively (Tables 3 and 4). In addition, there were 17 PS-unique proteins such as MAPK14 (mitogen-activated protein kinase 14), MAP2K1 (mitogen-activated protein kinase kinase 1), HECTD1 (HECT-type E3 ubiquitin transferase), HSD17B2 (estradiol 17-beta-dehydrogenase 2), HPGD (15-hydroxyprostaglandin dehydrogenase), RPS6 (40S ribosomal protein S6) and DLG1 (discs large MAGUK scaffold protein 1), which were found to play important roles in reproductive processes, specifically in relation to the establishment and maintenance of pregnancy (Table 5). The functional analysis of the unique proteins that were identified in the NPS endometrium samples, revealed that 58, 25, and 25 proteins were involved in immune system process, biological adhesion and reproductive process, respectively. It was observed that some of these proteins played important roles in the development of the reproductive cycle (Tables 6, 7 and 8).

**Table 3:** Complete list of exclusive proteins involved in immune system process in the endometrium of pregnant sows (PS) during implantation

Entry	Protein name	Gene symbol
W5PXV8	40S ribosomal protein S6	*RPS6*
W5NZ21	ATPase family AAA domain containing 3A	*LOC101105090*
S9XCQ7	BCL2-associated athanogene 6	*CB1_000383021*
Q6YT41	C-C chemokine receptor type 5	*CCR5*
F1RUL6	C-X-C motif chemokine	*LOC100520680*
A0A286ZJQ9	Caveolin	*CAV1*
Q0KIZ6	CD276 antigen	*Cd276*
Q764N1	CD74 antigen	*CD74 cd74*
A0A1S3WDL1	Complement C3	*LOC103115217*
F1S788	Complement C8 alpha chain	*C8A*
L9KLP8	Complement component 1 Q subcomponent-binding protein, mitochondrial	*TREES_T100004043*
A0A287BG06	Cytochrome b(558) alpha chain	*CYBA*
G3TC02	Discs large MAGUK scaffold protein 1	*DLG1*
I3M958	DNA mismatch repair protein	*MSH2*
F1PAK1	Dynamin GTPase	*DNM1*
F1SI48	Erythrocyte membrane protein band 4.2	*EPB42*
F1RGS2	Glucosylceramidase	*GBA*
F1RUE4	Glycosyl-phosphatidylinositol-specific phospholipase D	*GPLD1*
G7Q1J3	Haptoglobin	*EGM_11911*
W5P7R2	HECT-type E3 ubiquitin transferase	*HECTD1*
P32394	Heme oxygenase 1	*HMOX1*
O98263	HLA class II histocompatibility antigen, DQ beta 1 chain isoform 1	*SLADQB*
A0A088CPQ8	HLA class II histocompatibility antigen, DRB1-4 beta chain isoform X2	*SLA-DRB1*
I3LGI4	Interferon induced protein 35	*IFI35*
A0A287AB58	ISG15 ubiquitin like modifier	*ISG15*
W5PQ26	KDEL endoplasmic reticulum protein retention receptor 1	*KDELR1*
B3F714	Lipopolysaccharide-binding protein	*LBP*
A1YH82	MHC class I antigen	*SLA-2*
Q0MRZ9	MHC class I antigen	*SLA*
Q7YQ94	MHC class II antigen	*SLA-DQA1*
Q9XS06	MHC class II antigen	*Odvi-DRB*
Q1W6C6	MHC class II antigen DM	*SLA-DMB*
U3EP06	Mitogen-activated protein kinase 14	*MAPK14*
W5Q9X5	Mitogen-activated protein kinase kinase 1	*MAP2K1*
A2SW51	Monocyte differentiation antigen CD14	*CD14*
G1L645	Natural resistance-associated macrophage protein 1	*SLC11A2*
U6CWZ8	NFX1-type zinc finger-containing protein 1	*ZNFX1*
W5Q3A5	Platelet-activating factor acetylhydrolase IB subunit alpha	*PAFAH1B1*
M3XVA8	Protein kinase C	*PKN1*
H2Q026	Protein S100	*S100A9*
P31151	Protein S100-A7	*S100A7*
H2QN65	RAB, member of RAS oncogene family-like 3	*RABL3*
W5PDR1	RAB6A, member RAS oncogene family	*RAB6A*
F1RS76	Raftlin, lipid raft linker 1	*RFTN1*
W5PLQ9	Ribosome maturation protein SBDS	*SBDS*
A0A287BKR2	Serine	*SHMT2*
G3SQP8	SH2B adaptor protein 2	*SH2B2*
M3Z0V1	SIN3 transcription regulator family member A	*SIN3A*
W5PUJ4	Synaptotagmin binding cytoplasmic RNA interacting protein	*SYNCRIP*
F1RYZ1	Tetraspanin	*CD151*
H0XH45	Uncharacterized protein	*NCKAP1L*
H0XWE2	Uncharacterized protein	*STXBP2*
W5P0S1	Vesicle associated membrane protein 7	*VAMP7*

**Table 4:** Complete list of exclusive proteins involved in biological adhesion annotation in the endometrium of pregnant sows (PS) during implantation

Entry	Protein name	Gene symbol
G1LXE3	Actinin alpha 3	*ACTN3*
C3VPJ4	Claudin	*CLDN7*
A0A212DIV7	CTNND1	*Celaphus_00009417*
A0A287BJI7	Cytochrome P450 1B1	*CYP1B1*
A0A2C9F3H7	Dipeptidyl peptidase 4	*DPP4*
G3TC02	Discs large MAGUK scaffold protein 1	*DLG1*
E1BLS8	Elastin microfibril interfacer 1	*EMILIN1*
A0A1S3G8T8	fibrinogen gamma chain	*Fgg*
Q5D862	Filaggrin-2	*FLG2 IFPS*
F1REZ1	Hyaluronan and proteoglycan link protein 1	*HAPLN1*
L5JSB3	Integrin alpha-3	*PAL_GLEAN10019711*
T0NP01	Integrin beta	*CB1_000265043*
S9XMV3	Junctional adhesion molecule 1	*CB1_000141016*
G1RRE5	Leupaxin	*LPXN*
L9L235	Myosin-6	*TREES_T100007155*
H2PHD0	Non-specific serine/threonine protein kinase	*STK10*
G3WCH8	Periostin	*POSTN*
H2Q026	Protein S100	*S100A9*
W5QDT4	Ras homolog family member B	*RHOB*
A0A287B6S0	Sorbin and SH3 domain containing 1	*SORBS1*
I3LUI4	Tenascin	*TNC*
F1RHA7	Transforming growth factor-beta-induced protein ig-h3	*TGFBI*
F1RIS5	VWFA domain-containing protein	*ITGAM*

**Table 5:** Complete list of exclusive proteins with reproductive process annotation in the endometrium of pregnant sows (PS) during implantation

Entry	Protein name	Gene symbol	Reproductive protein function
X5D8S1	(S)-3-amino-2-methylpropionate transaminase	*ABAT*	Copulation
D0G6X9	15-hydroxyprostaglandin dehydrogenase	*HPGD*	Female pregnancy, ovulation, parturition
W5PXV8	40S ribosomal protein S6	*RPS6*	Gastrulation, placenta development, mammalian oogenesis stage
U3DRY3	Activin beta-B chain	*INHBB*	Oocyte development, regulation of ovulation
S9XCQ7	BCL2-associated athanogene 6	*CB1_000383021*	Spermatogenesis
H0X9I7	Diaphanous related formin 2	*DIAPH2*	Female gamete generation
G3TC02	Discs large MAGUK scaffold protein 1	*DLG1*	Embryonic skeletal system, reproductive structure development
I3M958	DNA mismatch repair protein	*MSH2*	Utero embryonic development, germ cell development
A0A287BB42	Estradiol 17-beta-dehydrogenase 2	*HSD17B2*	Utero embryonic development, placenta development, response to retinoic acid
W5P7R2	HECT-type E3 ubiquitin transferase	*HECTD1*	Trophoblast giant cell diferenciation, spongiotrophoblast differenciation
L9KSA3	Maillard deglycase	*TREES_T100005313*	Single fertilization
U3EP06	Mitogen-activated protein kinase 14	*MAPK14*	Placenta development
W5Q9X5	Mitogen-activated protein kinase kinase 1	*MAP2K1*	Placenta blood vessel development
E2RNV3	PITH domain containing 1	*PITHD1*	Penetration of cumulus oophorus and zona pellucida
S7MGV6	Splicing factor 3A subunit 2	*D623_10016373*	Gonad development
W5PQF0	T-complex protein 1 subunit eta	*CCT7*	Binding of sperm to zona pellucida
A0A287AMZ2	T-complex protein 1 subunit gamma	*CCT3*	Binding of sperm to zona pellucida

**Table 6:** Complete list of exclusive proteins involved in immune system process in the endometrium of non-pregnant sows (NPS) at d 18 and 24 of cycle

Entry	Protein name	Gene symbol
W5PEV6	40S ribosomal protein S19	*LOC101107920*
H0UYD9	75 kDa glucose-regulated protein (Heat shock 70 kDa protein 9)	*HSPA9*
Q2VI03	92 kDa gelatinase	*MMP9*
W5Q3F7	Abl interactor 1	*ABI1*
I3L704	Anamorsin (Cytokine-induced apoptosis inhibitor 1)	*CIAPIN1*
P49931	Antibacterial peptide PMAP-36	*PMAP36*
Q8HYU5	Apoptosis regulator Bax	*BAX*
W5PBA2	BCL2-associated athanogene 6	*BAG6*
H2R533	Butyrophilin like 9	*BTNL9*
A0A287BRD6	C–C motif chemokine	*CCL16*
I3LUE7	C5a anaphylatoxin chemotactic receptor	*C5AR1*
W5PRC4	Calcium and integrin-binding protein 1	*CIB1*
K7GPF5	CD248 molecule	*CD248*
W5QBV7	CD44 antigen	*CD44*
B0LUW3	Chemerin	*RARRES2*
T0MGW0	Complement C3-like protein	*CB1_000418018*
P51779	Complement factor D	*CFD*
K7GQR1	Complement factor properdin	*CFP*
U6CV23	DNA polymerase	*DPOLB*
M3XQV3	E3 UFM1-protein ligase 1	*UFL1*
I3LK80	Elastase, neutrophil expressed	*ELANE*
F1S2R7	Eukaryotic translation initiation factor 2B subunit beta	*EIF2B2*
I3NEL2	Exosome component 6	*EXOSC6*
K7GKS3	FAD-binding FR-type domain-containing protein	*GP91-PHOX*
Q765P8	Glutathione-independent PGD synthase	*Pgds*
G3SHS4	Guanine nucleotide exchange factor H1	*ARHGEF2*
A0A250YG48	Haptoglobin	*HP*
X5D945	Harvey rat sarcoma viral oncoprotein isoform A	*HRAS*
A0A0A1E9A8	Immmunoglobulin lambda light chain variable region	*IGL*
H2RAG8	Immunoglobulin kappa variable 1D-43	*IGKV1D-43*
F1SC98	Insulin degrading enzyme	*IDE*
F1RRC1	Integrin alpha-9	*ITGA9*
Q29056	Interleukin-1 receptor antagonist protein	*IL1RN*
K7GPB4	Mannose receptor C-type 1	*MRC1*
O19245	MHC class I antigen	*PD14*
Q8MGY0	MHC class I antigen	*SLA-P1*
Q8MGX5	MHC class II antigen	*SLA-DQA*
F1P7V2	Myeloid differentiation primary response protein MyD88	*MYD88*
A0A287AE25	Non-secretory ribonuclease	*LOC102163838*
F1S3Q7	Pantetheinase	*VNN1*
M3XNB8	Peptidyl-prolyl cis-trans isomerase	*PPIB*
A0A286ZND5	Peroxiredoxin-1	*PRDX1*
W5Q3A5	Platelet-activating factor acetylhydrolase IB subunit alpha	*PAFAH1B1*
P79281	Pleiotrophin	*PTN*
W5PPM6	Polyglutamine binding protein 1	*PQBP1*
H0X102	Proteasome subunit beta	*PSMB6*
A0A1U7TYL3	protein jagunal homolog 1	*JAGN1*
A0A061I0D8	Putative interferon-induced protein like protein	*H671_7g18688*
G3TQR7	RAB43, member RAS oncogene family	*RAB43*
G5AUH7	Ras-related protein Rab-3B	*GW7_07140*
A0A091D8J0	Receptor protein-tyrosine kinase	*H920_19434*
M3XWF9	RNA binding motif protein 14	*RBM14*
A0A091CPP3	RNA helicase	*H920_17538*
A7LCX1	RNA helicase	*MDA5*
Q5U3Z7	Serine hydroxymethyltransferase	*Shmt2*
F7HJP2	Spectrin alpha	*SPTA1*
W5PES0	Syntaxin 4	*STX4*
W5NYY0	Translation initiation factor eIF-2B subunit alpha	*EIF2B1*

**Table 7:** Complete list of exclusive proteins involved in biological adhesion annotation in the endometrium of non-pregnant sows (NPS) at d 18 and 24 of cycle

Entry	Protein name	Gene symbol
F1RY44	Cadherin 17	*CDH17*
Q28984	CD11b (Fragment)	*CD11b*
M3YTD5	Cellular communication network factor 2	*CCN2*
F1SAT8	Complement component C1q receptor	*CD93*
A0A091DIG4	Dystonia 1 protein (Torsin-1A)	*H920_06675*
K7GQN2	Fibroblast activation protein alpha	*FAP*
A0A250Y8E7	Fibronectin	*FN1*
F1RRC1	Integrin alpha-9	*ITGA9*
K9IVW2	Integrin alpha-X	*ITGAX*
A0A0R7F1J7	Integrin beta	*ITGB4*
K7GKU7	Integrin subunit alpha 3	*ITGA3*
F1SBB3	Laminin subunit alpha 3	*LAMA3*
K7D2I3	Laminin, alpha 5	*LAMA5*
F1S3Q7	Pantetheinase	*VNN1*
W5PEP0	Parvin alpha	*PARVA*
F1S4A6	PDZ domain-containing protein	*SYNJ2BP*
W5Q0M7	Ras-related protein Rap-2	*RAP2B*
W5PWC5	Receptor protein-tyrosine kinase	*EGFR*
F1SPK8	Tetraspanin	*CD63*
I3L8H4	Thrombospondin 4	*THBS4*
F1S7Q6	Tight junction protein 3	*TJP3*
S9X0Z8	Transforming growth factor-beta-induced protein ig-h3	*CB1_000686008*
K7GRK7	Uncharacterized protein	*TNXB*
A0A287BHG0	Uncharacterized protein	*NRCAM*
Q29123	Vascular cell adhesion molecule	*VCAM*

**Table 8:** Complete list of exclusive proteins with reproductive process annotation in the endometrium of non-pregnant sows (NPS) at d 18 and 24 of cycle

Entry	Protein name	Gene symbol	Reproductive protein function
G3STE1	72 kDa gelatinase	*MMP2*	Embryo implantation
A0A287BPU6	Adenylate cyclase 3	*ADCY3*	Flagellated sperm motility
Q8HYU5	Apoptosis regulator Bax	*BAX*	Fertilization, ovarian follicle development, regulation of cell cycle
M3XLY3	ATP-dependent helicase ATRX	*ATRX*	Meiotic spindle organization, spermatogenesis, sertoli cell development
W5PBA2	BCL2-associated athanogene 6	*BAG6*	Spermatogenesis
W5PRC4	Calcium and integrin-binding protein 1	*CIB1*	Spermatid development
F1S2R7	Eukaryotic translation initiation factor 2B subunit beta	*EIF2B2*	Ovarian follicle development
J9JIM2	Glutathione hydrolase	*GGT1*	Response to estradiol, spermatogenesis
W5Q6F0	Histone H2A	*LOC101106791*	Spermatogenesis
Q8MKG1	Hydroxysteroid 11-beta dehydrogenase 2	*HSD11B2*	Female pregnancy
I3LEF8	Hydroxysteroid 17-beta dehydrogenase 4	*HSD17B4*	Sertoli cell development, estrogen metabolic process
H0WMK3	Intraflagellar transport 20	*IFT20*	Spermatogenesis
F1SKJ7	Intraflagellar transport 27	*IFT27*	Spermatogenesis
A0A287AMZ5	Membrane cofactor protein	*CD46*	Single fertilization
W5P0A6	Platelet activating factor acetylhydrolase 1b catalytic subunit 2	*PAFAH1B2*	Spermatogenesis
P79281	Pleiotrophin	*PTN*	Estrous cycle, oogenesis
Q5PQN1	Probable E3 ubiquitin-protein ligase HERC4	*Herc4*	Spermatogenesis
F1SV76	Progesterone receptor	*PGR*	Ovulation from ovarian follicle
L5KWA4	Protein diaphanous like protein 3	*PAL_GLEAN10008668*	Female gamete generation
I3LDE6	Rhophilin associated tail protein 1 like	*ROPN1L*	Sperm capacitation
G5E704	Ribosomal protein L10 like	*RPL10L*	Spermatogenesis
M3WPZ7	UV excision repair protein RAD23	*RAD23B*	Spermatogenesis
F1S8D4	WD repeat domain 77	*WDR77*	Prostate gland development

#### Network interaction of identified PS-unique proteins

**Fig. 3:**
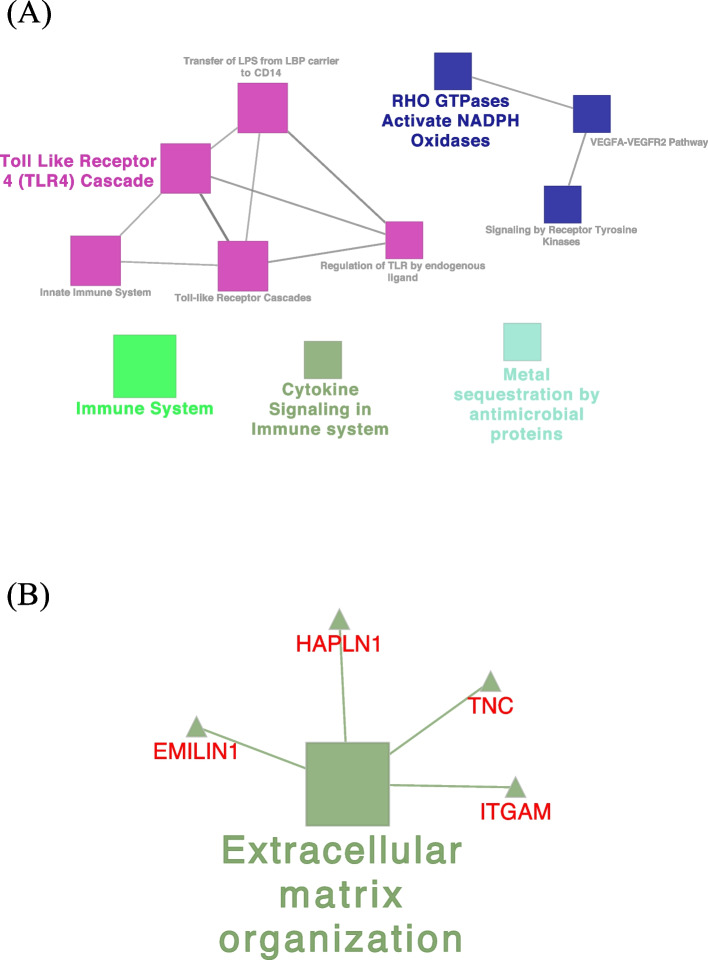
Visualization of the reactome pathways analysis of unique proteins involved in immune system process (**A**) and biological adhesion annotation (**B**) in pregnant sows. The size of the nodes indicates the pathway´s enrichment significance. The following ClueGO parameters were used: *P*-value cut-off = 0.05; Statistical test used the enrichment/depletion (two-sided hypergeometric test); Bonferroni step down with minimum GO level 2; maximum GO level 6; kappa score threshold equal to 0.4. GO = Gene Ontology. Network (**A**) includes the interaction of CCR5, HMOX1, IFI35, ISG15, LBP, MAP2K1, MAPK14, SLA, STXBP2, VAMP7, CD14, DNM1, LBP, MAP2K1, MAPK14 and S100A9 proteins

Analysis of the PS-unique proteins, using ClueGO Pathway Enrichment Analysis, revealed an interaction network of proteins involved in immune system process in 11 REACTOME pathways. Among them, the interaction of CCR5 (C-C chemokine receptor type 5), HMOX1 (heme oxygenase 1), IFI35 (interferon induced protein 35), ISG15 (ISG15 ubiquitin like modifier), LBP (lipopolysaccharide-binding protein), MAP2K1, MAPK14, SLA (MHC class I antigen), *STXBP2* (uncharacterized protein), VAMP7 (vesicle associated membrane protein 7) proteins in the network of cytokine signaling in immune system and CD14 (monocyte differentiation antigen CD14), DNM1 (dynamin GTPase), LBP (lipopolysaccharide-binding protein), MAP2K1, MAPK14, S100A9 (protein S100) proteins in the network of Toll Like Receptor 4 cascade pathways (Fig. 3A). The analysis also revealed the interaction of EMILIN1 (elastin microfibril interfacer 1), HAPLN1 (hyaluronan and proteoglycan link protein 1), ITGAM (VWFA domain-containing protein), *TNC* (tenascin) proteins involved in biological adhesion annotation in the extracellular matrix organization REACTOME pathway (Fig. 3B). Regarding the identified PS-unique proteins involved in reproductive process annotation, ClueGO showed the interaction of DLG1, MAP2K1, MAPK14 proteins in T cell receptor signaling KEGG pathway and CCT3 (T-complex protein 1 subunit gamma), CCT7 (T-complex protein 1 subunit eta) proteins in Folding of actin by CCT/TriC REACTOME pathway.

**Fig. 4:**
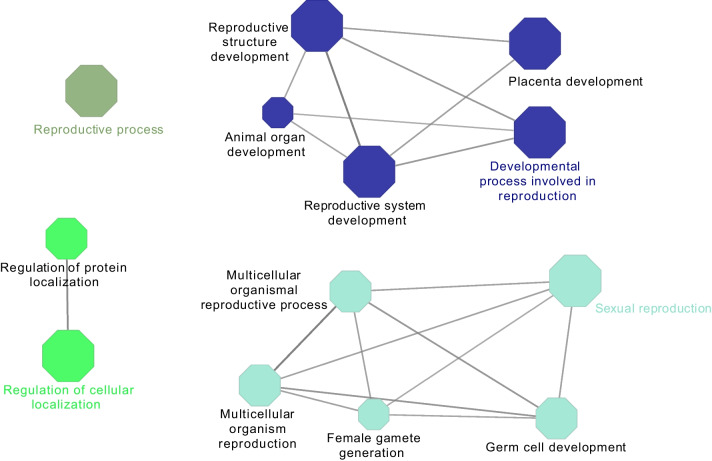
Visualization of the biological gene ontology analysis of unique proteins involved in the reproductive process annotation in pregnant sows. The size of the nodes indicates the pathway´s enrichment significance. The following ClueGO parameters were used: *P*-value cut-off = 0.001; Statistical test used the enrichment/depletion (two-sided hypergeometric test); Bonferroni step down with minimum GO level 2; maximum GO level 6; kappa score threshold equal to 0.4. GO = Gene Ontology. Network include the involvement of HECTD1, HSD17B2, MAP2K1, MAPK14, RPS6, MSH2, DLG1 and INHBB proteins among others

Cytoscape ClueGO analysis also revealed the involvement of PS-unique proteins with reproductive process annotation in various GO terms of Biological Process (Fig. 4) such as placenta development (HECTD1, HSD17B2, MAP2K1, MAPK14, RPS6) or reproductive structure and system development (HECTD1, HSD17B2, MAP2K1, MAPK14, RPS6, MSH2 (DNA mismatch repair protein), DLG1, INHBB (activin beta-B chain)).

Supplementary information on the proteins belonging to each pathway can be found in Tables S6–S9.

#### Network interaction of identified NPS-unique proteins

**Fig. 5:**
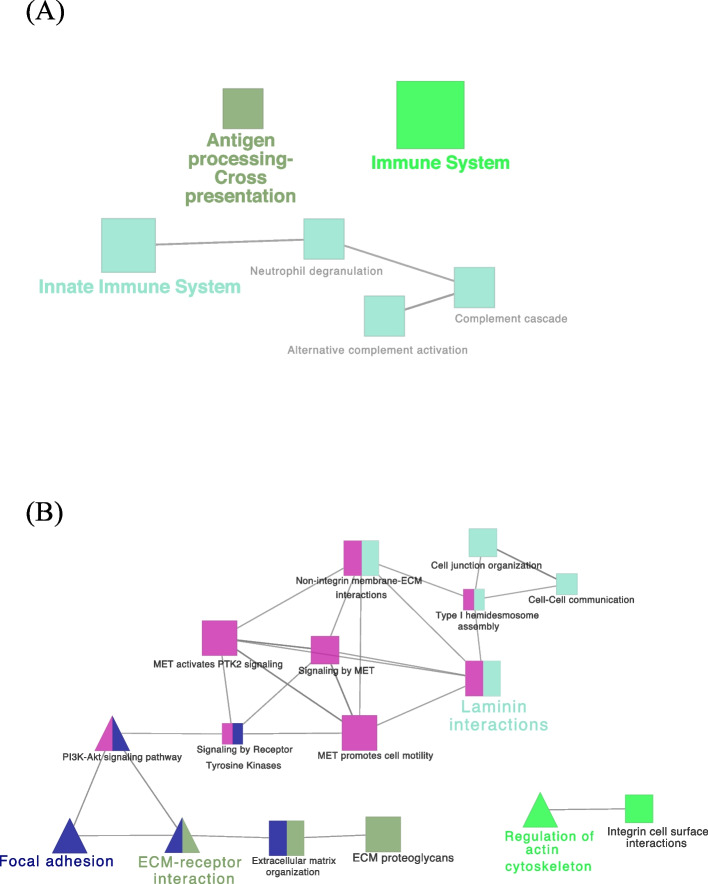
Visualization of the reactome pathways analysis of unique proteins involved in immune system process (**A**) and in biological adhesion annotation (**B**) in non-pregnant sows. The size of the nodes indicates the pathway´s enrichment significance. The following ClueGO parameters were used: *P*-value cut-off = 0.05; Statistical test used the enrichment/depletion (two-sided hypergeometric test); Bonferroni step down with minimum GO level 2; maximum GO level 6; kappa score threshold equal to 0.4. GO = Gene Ontology. Network (**A**) includes the interaction of ABI1, C5AR1, CD44, CFD, CFP, ELANE, HP, HRAS, IFIH1, MMP9, MYD88, PSMB6 and VNN1 proteins. Network (**B**) includes the interaction of FN1, ITGA3, ITGA9, ITGB4, LAMA3, LAMA5, THBS4, TNXB, EGFR, ITGAX, EGFR, PARVA, and TNXB proteins

**Fig. 6:**
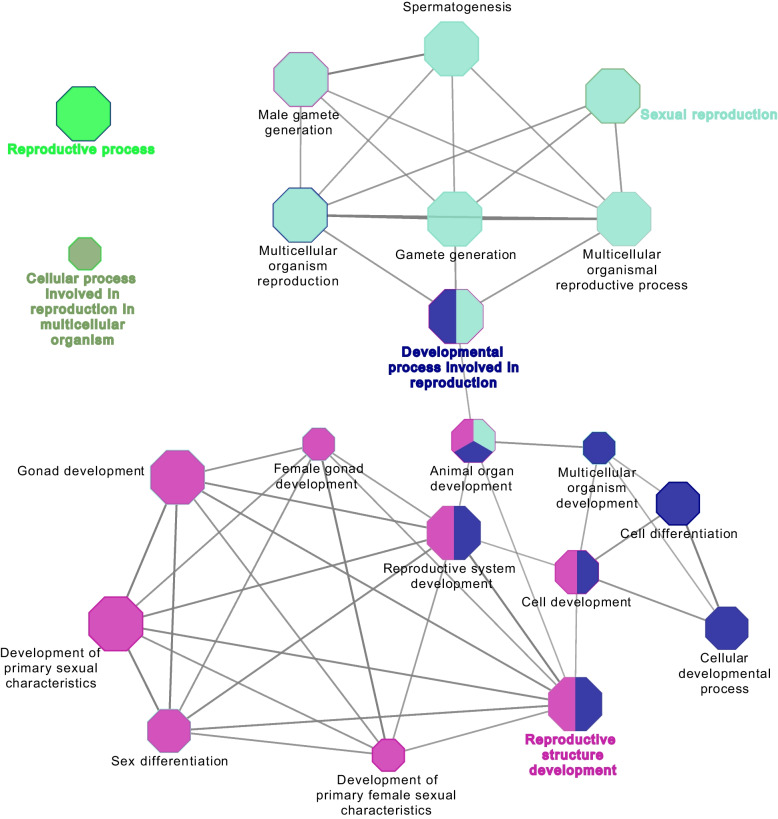
Visualization of the biological gene ontology analysis of unique proteins involved in the reproductive process annotation in non-pregnant sows. The size of the nodes indicates the pathway’s enrichment significance. The following ClueGO parameters were used: *P*-value cut-off = 0.001; Statistical test used the enrichment/depletion (two-sided hypergeometric test); Bonferroni step down with minimum GO level 2; maximum GO level 6; kappa score threshold equal to 0.4. GO = Gene Ontology

Analysis of the NPS-unique proteins, using ClueGO analysis, revealed an interaction network of proteins (ABI1 (Abl interactor 1), C5AR1 (C5a anaphylatoxin chemotactic receptor), CD44 (CD44 antigen), CFD (complement factor D), CFP (complement factor properdin), ELANE (elastase, neutrophil expressed), HP (haptoglobin), HRAS (Harvey rat sarcoma viral oncoprotein isoform A), MMP9 (92 kDa gelatinase), MYD88 (myeloid differentiation primary response protein MyD88), PSMB6 (proteasome subunit beta) and VNN1 (pantetheinase)) involved in immune system in the innate immune system REACTOME pathway (Fig. 5A, Table S10). Functional ClueGO enrichment analysis of the NPS-unique proteins involved in biological adhesion revealed the interaction of FN1 (fibronectin), ITGA3 (integrin subunit alpha 3), ITGA9 (integrin alpha-9), ITGB4 (integrin beta), LAMA3 (laminin subunit alpha 3), LAMA5 (laminin, alpha 5), THBS4 (thrombospondin 4) and TNXB (uncharacterized) proteins network in ECM-receptor interaction, EGFR (receptor protein-tyrosine kinase), FN1, ITGA3, ITGA9, ITGAX (integrin alpha-X) and ITGB4 proteins network in regulation of actin cytoskeleton, and EGFR, FN1, ITGA3, ITGA9, ITGB4, LAMA3, LAMA5, PARVA (parvin alpha), THBS4 and TNXB proteins network in focal adhesion KEGG pathways (Fig. 5B, Table S11). In addition, ClueGO analysis of the NPS-unique proteins involved in the reproductive process showed that most proteins were functionally enriched in multiple GO terms (Fig. 6, Table S12) with proteins involved in reproductive functions related to different stages of the estrous cycle such as fertilization, ovarian follicle development, regulation of cell cycle, ovarian follicle development, response to estradiol, estrous cycle, oogenesis and ovulation from the ovarian follicle.

### Analysis of the extraembryonic membrane proteome profile

The UniProt mammalian library with FDR correction allowed the identification of 3,968 proteins in extraembryonic membranes of PS during the peri-implantation period (d 18 and 24 of gestation). The list of all identified proteins, including their unused score, percentage of sequence coverage, UniProt accession number, protein name, species and matched peptides can be found in Table S13. Table 9 shows the ten most highly abundant proteins identified in the extraembryonic membranes. This group included some critical extracellular matrix proteins, such as fibronectin, which is a multifunctional adhesive glycoprotein with a relevant role in embryogenesis, and heparan sulfate proteoglycan 2, which interacts with many other proteins and has many functions in cell signaling, adhesion and angiogenesis. Other proteins involved in cytokinesis and cell shape maintenance, such as myosin 9, filamin A, and plectin were also present. Other proteins found in this group act as carrier proteins and bind to numerous growth factors and cytokines, such as alpha-2 macroglobulin. Additionally, proteins involved in cell differentiation, including collagen type XII alpha 1 chain and if rod domain-containing protein, are also involved in embryonic placental development.

**Table 9:** Summary of high abundance proteins in extra-embryonic membranes of pregnant sows (PS) during implantation

Protein name	Gene symbol	UniProt ID	Molecular function	Biological process	Matched peptides
Fibronectin	*FN1*	F1SS24_PIG	Enables binding	Involved in interaction, blood coagulation, cell matrix adhesion	441
Fibronectin	*FN1*	A0A286ZY95_PIG	Enables heparin binding	Involves in cell adhesion and regulation of cell shape	429
Heparan sulfate proteoglycan 2	*HSPG2*	F1SU03_PIG	Enables binding to a calcium ion		274
Myosin-9	*MYH9*	K9IVP5_PIG	Enables cytoskeletal motor activity, ATP binding, actin filament binding	Involved in phagocytosis	254
IF rod domain-containing protein	*KRT14*	F1S0J8_PIG	Enables structural molecule activity	Involved in response to estrogen and cell differentiation involved in embryonic placenta development	253
Hemoglobin subunit epsilon	*HBE1*	F1RII6_PIG	Enables metal ion binding, oxygen binding, hemo binding, organic acid binding	Involved in oxygen transport and cellular oxidant detoxification	242
Filamin A	*FLNA*	A0A286ZXU2_PIG	Enables actin binding	Involved in actin cytoskeleton organization	228
Alpha-2-macroglobulin	*A2M*	K9J6H8_PIG	Enables peptidase inhibitor activity	Involved in negative regulation of endopeptidase activity	225
Collagen type XII alpha 1 chain	*COL12A1*	F1RQI0_PIG		Involved in endodermal cell differenciation	217
Plectin	*PLEC*	A0A287BNK7_PIG	Enables cytoskeleton protein binding and actin binding	Involved in intermediate filament cytoskeleton organization	200

**Fig. 7:**
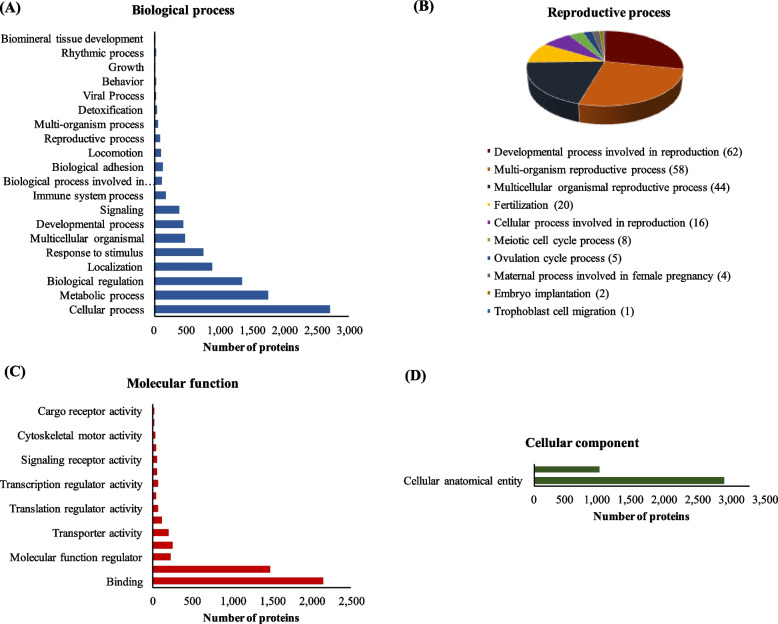
Gene Ontology analysis of the extraembryonic membranes proteome of pregnant sows at d 18 and 24 of gestation. **A** Biological process. **B** Reproductive process. **C** Molecular function. **D** Cellular component

Of the proteins identified in the extraembryonic membranes, a total of 3,808 proteins were successfully mapped to UniProtKB IDs, with 3,114, 3,014 and 3,109 proteins annotated with biological process, molecular function and cellular component annotation, respectively (Fig. 7). Within the proteins that were annotated with biological process, 132 proteins were involved with biological adhesion annotation (Table 10) and 94 proteins were associated with reproductive process (Fig. 7B, Table 11).

**Table 10:** Complete list of extraembryonic membranes proteins of pregnant sows (PS) involved in biological adhesion annotation

Entry	Protein name	Gene symbol
Q8SQC1	Scavenger receptor class B member 1	*SCARB1*
Q8WNW3	Junction plakoglobin	*Jup*
P26234	Vinculin	*VCL*
Q62812	Myosin-9	*Myh9*
P05027	Sodium/potassium-transporting ATPase subunit beta-1 (	*ATP1B1*
B5B2Z3	Alpha v integrin subunit (Integrin alpha-V isoform 1 preproprotein)	*ITGAV*
U6CR38	Alpha-parvin	*PARVA*
U6CN56	Cyclin-dependent kinase 5	*CDK5*
F1SAT8	Complement component C1q receptor	*CD93*
F1SI18	Frizzled class receptor 7	*FZD7*
F1MMN6	Integrin subunit alpha 1	*ITGA1*
W5Q731	59 kDa serine/threonine-protein kinase (Beta-integrin-linked kinase)	*ILK*
W5Q5R2	Neural cell adhesion molecule 1	*NCAM1*
W5PXV3	Cellular communication network factor 2	*CCN2*
W5QD08	Laminin subunit gamma 1	*LAMC1*
W5PNV2	Serine/threonine-protein phosphatase (EC 3.1.3.16)	*PPP1CA*
W5NQS2	Integrin subunit alpha 9	*ITGA9*
F1MEG3	Laminin subunit alpha 1	*LAMA1*
W5NY24	Catenin beta 1	*CTNNB1*
A0A286XCN5	Ninjurin 1	*NINJ1*
A0A1U8BRS2	Protocadherin Fat 1 isoform X1	*Fat1*
I3MKW0	Glypican 4	*GPC4*
G3HLU9	Junction plakoglobin	*I79_011694*
G3I1V3	Fibronectin	*I79_017372*
A0A1S3FZT4	Talin-1 isoform X2	*Tln1*
Q8C7Q6	Uncharacterized protein	*Cdh11*
G3HN02	Talin-1	*I79_012132*
A0A091D8T9	Myosin-6	*H920_11044*
I3NCN5	Heat shock protein 90 alpha family class B member 1	*HSP90AB1*
A0A1S3FB44	Fibronectin	*Fn1*
A0A212D5I5	DSP (Fragment)	*Celaphus_00014627*
T0NP01	Integrin beta	*CB1_000265043*
A0A1S3WT97	Integrin beta	*ITGB1*
L9KGM3	Oxysterol-binding protein (Fragment)	*TREES_T100009169*
L5JXS8	Catenin alpha-1	*PAL_GLEAN10016813*
S7N5C4	Calsyntenin-1	*D623_10021980*
A0A212D3W3	Fibronectin	*Celaphus_00015077*
F1SKU3	Crumbs cell polarity complex component 2	*CRB2*
F1SMF6	Integrin subunit alpha 1	*ITGA1*
A0A2I3GKG9	Fibronectin	*FN1*
K9IIB4	40S ribosomal protein SA (37 kDa laminin receptor precursor) (37LRP)	*RPSA*
A0A287AYB4	Platelet-derived growth factor receptor alpha	*PDGFRA*
K7GSR6	Ecto-5'-nucleotidase (EC 3.1.3.5)	*NT5E*
F1SS24	Fibronectin	*FN1*
K7GPI3	Platelet endothelial cell adhesion molecule	*PECAM1*
A0A287AG36	Laminin subunit alpha 1	*LAMA1*
A7YX23	Torsin	*DYT1*
Q9TUN5	Integrin beta	*GPIIIa*
F1SJY6	Catenin delta 1	*CTNND1*
I3LUR7	Collagen type VI alpha 3 chain	*COL6A3*
B3TFD9	Nectin cell adhesion molecule 2	*NECTIN2*
C3VML0	Claudin	*CLDN4*
C3S7K6	Calcium-binding protein A9	
B2ZI35	Junctional adhesion molecule 1	
F1RGD0	RIC8 guanine nucleotide exchange factor A	*RIC8A*
B9ZSM8	Thy-1 antigen	*THY1 CD90*
I3LTD0	Lymphatic vessel endothelial hyaluronan receptor 1	*LYVE1*
C3S7K5	Protein S100 (S100 calcium-binding protein)	*S100A8*
F1RZL4	ADAM metallopeptidase domain 9	*ADAM9*
F1SFE3	Fermitin family homolog 2 isoform X1	*FERMT2*
Q9MZU5	Intercellular adhesion molecule 1	*ICAM-1*
A8DSD5	Integrin beta	
F1REZ1	Hyaluronan and proteoglycan link protein 1	*HAPLN1*
F1SPK8	Tetraspanin	*CD63*
F1SR53	Integrin alpha-5	*ITGA5*
B0LY42	Basigin	
Q8WNW5	Cadherin-5	*CDH5*
C3VML2	Claudin	*CLDN6*
K7GPD4	Integrin beta	*ITGB4*
F1RS37	Periostin	*POSTN*
F1SFH7	Uncharacterized protein	*LPP*
V5L1C3	POSTN	*POSTN*
K7GNN0	von Willebrand factor (vWF)	*VWF*
F1SMF4	Integrin subunit alpha 2	*ITGA2*
A0A287A3D8	CD34 molecule	*CD34*
K7GRK7	Uncharacterized protein	*TNXB*
A0A2J8Q3M0	Protein S100 (S100 calcium-binding protein)	*S100A8*
A0A287AC34	Talin 1	*TLN1*
I3LDQ1	Uncharacterized protein	*TLN2*
F1RW75	Desmoplakin	*DSP*
I3LUI4	Tenascin	*TNC*
F1SS26	Thrombospondin 1	*THBS1*
F1RGP4	Uncharacterized protein	*PBXIP1*
G3FNU5	Integrin beta	
F1S663	Laminin subunit gamma 1	*LAMC1*
K7GSP7	Discs large MAGUK scaffold protein 1	*DLG1*
Q28939	Vascular cell adhesion molecule	
K7GND9	CD44 antigen	*CD44*
K7GKU7	Integrin subunit alpha 3	*ITGA3*
F1SKM1	Uncharacterized protein	*COL7A1*
K7GQN2	Fibroblast activation protein alpha	*FAP*
A0A287ACJ4	Integrin subunit alpha 4	*ITGA4*
Q0MSH8	Mucin 4 (Fragment)	*MUC4*
I3LFP3	Uncharacterized protein	*VCAN*
I3L638	Vitronectin	*VTN*
I3LJC9	Hyaluronan and proteoglycan link protein 3	*HAPLN3*
I3L5Z1	Cadherin-1 (Epithelial cadherin)	*CDH1*
F1S798	Endothelial cell adhesion molecule	*ESAM*
F1RGY5	Nidogen 1	*NID1*
F1RHA7	Transforming growth factor-beta-induced protein ig-h3	*TGFBI*
F1RZM4	Uncharacterized protein	*LAMA4*
F1SIC5	Neuroplastin	*NPTN*
I3LIM4	Desmoglein 2	*DSG2*
F1SBL4	Fermitin family member 1	*FERMT1*
F1S981	F-spondin (Spondin-1)	*SPON1*
A0A287AEV6	Uncharacterized protein	
Q0MS44	Presenilin (EC 3.4.23.-)	*PSEN1*
F1SBB3	Laminin subunit alpha 3	*LAMA3*
F1SIW0	Stabilin 1	*STAB1*
A8VKH9	Integrin-associated protein (Leukocyte surface antigen CD47)	
K7GT68	Integrin subunit alpha 6	*ITGA6*
I3L9I6	Basal cell adhesion molecule	*BCAM*
M3V7X9	Galectin-3-binding protein (Lectin galactoside-binding soluble 3-binding protein)	*LGALS3BP*
A0A2C9F3H7	Dipeptidyl peptidase 4	*DPP4*
F1RW86	Integrin subunit alpha 8	*ITGA8*
F1SAE9	Laminin subunit beta 1	*LAMB1*
I3LJW2	Fibrinogen gamma chain	*FGG*
F1SBY5	Afadin, adherens junction formation factor	*AFDN*
K7GQ83	Integrin beta	*ITGB1*
F1SNF3	Podocalyxin (Podocalyxin-like protein 1)	*PODXL*
F1SGD2	Plakophilin 2	*PKP2*
A0A286ZY95	Fibronectin	*FN1*
F1RRN0	Uncharacterized protein	*PTK7*
F1SB42	Ezrin	*EZR*
G3TXP4	Fermitin family member 3	*FERMT3*
K7D2I3	Laminin, alpha 5	*LAMA5*
M3WL90	Collagen type III alpha 1 chain	*COL3A1*
G3TBW6	Integrin subunit alpha M	*ITGAM*
U6DA73	Junctional adhesion molecule C (Fragment)	*JAM3*
F7BIL4	Integrin beta	*ITGB4*
L5KXI9	Paxillin	*PAL_GLEAN10010777*
L5KLH1	Integrin alpha-2	*PAL_GLEAN10009654*

**Table 11:** Complete list of extra-embryonic membranes proteins with reproductive process annotation

Entry	Protein name	Gene symbol	Reproductive protein function
M3Y3U2	Aladin WD repeat nucleoporin	*AAAS*	Fertilization
F1RRW5	Angiotensin-converting enzyme	*ACE*	Spermatogenesis
M3YH25	Actin-related protein 3	*ACTR3*	Meiotic cytokinesis
A0A287BPU6	Adenylate cyclase 3	*ADCY3*	Flagellated sperm motiligy
Q8MJ76	Alpha-fetoprotein	*AFP*	Protesterone metabolic process
F1SJB5	Annexin	*ANXA1*	Estrous cycle
A0A287AG13	Apolipoprotein B	*APOB*	Fertilization, in utero embryonic development, post-embryonic development, spermatogenesis
M3XLY3	DNA helicase	*ATRX*	Spermatogenesis
Q8HYU5	Apoptosis regulator Bax	*BAX*	Development of secondary sexual characteristics, fertilization, ovarian follicle development
F1S2A8	B-cell receptor-associated protein	*BCAP31*	Spermatogenesis
S9XCQ7	Large proline-rich protein BAG6	*CB1_000383021*	Spermatogenesis
A0A287AQV4	Cystathionine beta-synthase	*CBS*	Maternal process involved in female pregnancy
W5QBC9	Coiled-coil domain containing 134	*CCDC134*	Placenta development, embryonic hemopoiesis
A0A287AMZ2	T-complex protein 1 subunit gamma	*CCT3*	Binding of sperm to zona pellucida
F1SQN1	T-complex protein 1 subunit delta	*CCT4*	Binding of sperm to zona pellucida
I3LCA2	T-complex protein 1 subunit theta	*CCT8*	Binding of sperm to zona pellucida
W5PYM5	T-complex protein 1 subunit theta	*CCT8*	Binding of sperm to zona pellucida
A0A287AMZ5	Membrane cofactor protein	*CD46*	Single fertilization
Q8MJ48	Tetraspanin	*CD9*	Single fertilization
C0SW08	Cell division cycle 2	*CDK1*	Meiotic cell cycle process involved in oocyte maturation
F6SMS6	CUGBP Elav-like family member 1	*CELF1*	Spermatid development
A0A287ADH9	Chloride intracellular channel protein	*CLIC4*	Fertilization
F1SEB5	Ciliary neurotrophic factor receptor	*CNTFR*	Sex differenciation
F1RKX9	CRK like proto-onco, adaptor protein	*CRKL*	Single fertilization, spermatogenesis
A0A287AMJ5	Cartilage-associated protein	*CRTAP*	Spermatogenesis
W5NY24	Catenin beta 1	*CTNNB1*	Genitalia morphogenesis, ectoderm development, in utero embryonic development
Q29624	Aromatase 1	*CYP19A1*	Gonad development
M3VK01	RNA helicase	*DHX36*	Regulation of embryonic development
H0X9I7	Diaphanous related formin 2	*DIAPH2*	Female gamete generation
K7GSP7	Discs large MAGUK scaffold protein 1	*DLG1*	Reproductive structure development
H0W9W9	DnaJ heat shock protein family (Hsp40) member C19	*DNAJC19*	Genitalia development
F1S2R7	Eukaryotic translation initiation factor 2B subunit beta	*EIF2B2*	Ovarian follicle development
I3LEF1	eIF-2B GDP-GTP exchange factor subunit epsilon	*EIF2B5*	Ovarian follicle development
F1SFI6	Fetuin B (Fetuin-B isoform 1)	*FETUB*	Binding of sperm to zona pellucida
F1RI72	Golgin A3	*GOLGA3*	Spermatogenesis
P36968	Phospholipid hydroperoxide glutathione peroxidase	*GPX4*	Spermatogenesis
F6T4A1	HECT domain-containing protein	*HERC4*	Spermatogenesis
A0A287A7V3	Beta-hexosaminidase	*HEXA*	Sexual reproduction
D0G6X8	Beta-hexosaminidase	*HEXB*	Oogenesis, penetration of zona pellucida
Q8MKG1	Hydroxysteroid 11-beta dehydrogenase 2	*HSD11B2*	Female pregnancy
A0A287BB42	Estradiol 17-beta-dehydrogenase 2	*HSD17B2*	In utero embryonic development, placenta development
I3LEF8	Hydroxysteroid 17-beta dehydrogenase 4	*HSD17B4*	Sertoli cell development
I3NCN5	Heat shock protein 90 alpha family class B member 1	*HSP90AB1*	Placenta development
F7BIL4	Integrin beta	*ITGB4*	Cell adhesion, trophoblast cell migration
F7DLA5	Receptor protein-tyrosine kinase	*KDR*	Ovarian follicle development, embryonic hematopoiesis
W5NR48	Importin subunit alpha	*KPNA6*	Maternal process involved in female pregnancy
I3MMV6	Keratin 19	*KRT19*	Cell differentiation involved in embryonic placenta development
A0A1S3GYP8	Keratin, type I cytoskeletal 19	*Krt19*	Cell differentiation involved in embryonic placenta development
A0A1U7UCY2	Keratin, type I cytoskeletal 19 isoform X1	*KRT19*	Cell differenciation involved in embryonic placenta development
H2QCZ6	Keratin 9	*KRT9*	Spermatogenesis
F1S2F5	LHFPL tetraspan subfamily member 2	*LHFPL2*	Development of primary male and female sexual characteristics, positive regulation of fertilization
W5Q6F0	Histone H2A	*LOC101106791*	Spermatogenesis
C0HL13	Low-density lipoprotein receptor-related protein	*LRP2*	Gonad development
U3EP06	Mitogen-activated protein kinase 14	*MAPK14*	Placenta development
F1SQH4	DNA mismatch repair protein	*MSH2*	In utero embryonic development, male gonad development
Q62812	Myosin-9	*Myh9*	Utero embryonic development, follicle-stimulating hormone signaling pathway
F1SLS9	Condensin complex subunit 1	*NCAPD2*	Meiotic chromosome condensation
B3TFD9	Nectin cell adhesion molecule 2	*NECTIN2*	Acrosome assembly
G1LB85	Neurogenic locus notch homolog protein 1	*NOTCH1*	Prostate gland development
W5QGV9	Notch receptor 2	*NOTCH2*	Placenta blood vessel development, in utero embryonic development
F7FZQ6	Platelet-activating factor acetylhydrolase IB subunit alpha	*PAFAH1B1*	Germ cell development, positive regulation of embryonic development
Q0R678	Protein deglycase	*PARK7*	Single fertilization
F1RGP4	PBX homeobox interacting protein 1	*PBXIP1*	Spermatid nucleus differentiation
A0A287AYB4	Platelet-derived growth factor receptor alpha	*PDGFRA*	Utero embryonic development, luteinization, male genitalia development
I3LQN4	Prohibitin	*PHB2*	Mammary gland branching
H0VMB5	Phospholipid transfer protein	*PLTP*	Flagellated sperm motility
Q5XI34	Protein phosphatase 2	*Ppp2r1a*	Regulation of meiotic cell cyle process involved in oocyte maturation
F1S418	Peroxiredoxin 3	*PRDX3*	Maternal placenta development
W5P925	cAMP-dependent protein kinase	*PRKACA*	Sperm capacitation
H0X6Q0	Protein arginine N-methyltransferase	*PRMT7*	DNA methilation involved in gamete generation
U3DE75	Prostaglandin G/H synthase 2	*PTGS2*	Decidualization, embryo implantation
W5PIJ6	Tyrosine-protein phosphatase non-receptor type	*PTPN11*	Genitalia development
A0A0D9RIK4	Protein quaking	*QKI*	Spermatid development
M3WIX0	RAD21 cohesin complex component	*RAD21*	Meiosis I cell cycle process
F1SP32	UV excision repair protein RAD23	*RAD23B*	Spermatogenesis
Q6J1I8	E3 ubiquitin-protein ligase RNF114	*RNF114*	Spermatogenesis
W5Q9D9	RNA-splicing ligase RtcB homolog	*RTCB*	Placenta development, in utero embryonic development
H2QUF2	Septin	*SEPTIN7*	Spermatogenesis
F1SMW3	Serpin B5	*SERPINB5*	Prostate gland morphogenesis
W5PHB6	Splicing factor 1	*SF1*	Male sex determination, leydig cell differenciation
M3YZ90	Slit guidance ligand 2	*SLIT2*	Apoptotic process involved in luteolysis
H0VDS7	Mothers against decapentaplegic homolog	*SMAD1*	Gamete generation
W5PFJ5	Structural maintenance of chromosomes protein	*SMC1A*	Meiotic cell cycle
F1SRP0	Structural maintenance of chromosomes protein	*SMC2*	Meiotic cell cycle
W5PJ45	Structural maintenance of chromosomes protein	*SMC3*	Meiotic cell cycle
A0A287AT21	Sorbitol dehydrogenase	*SORD*	Flagellated sperm motility
A0A287AP26	ST14 transmembrane serine protease matriptase	*ST14*	Epithelial cell morphogenesis involved in placental branching
F1SB63	T-complex protein 1 subunit alpha	*TCP1*	Binding of sperm to zona pellucida
W5NVV3	Ubiquitin associated protein 2 like	*UBAP2L*	Binding of sperm to zona pellucida
F1S2F6	Voltage-dependent anion-selective channel protein 2	*VDAC2*	Binding of sperm to zona pellucida
F1S8D4	WD repeat domain 77	*WDR77*	Oocyte development
M3W8Q9	5'-3' exoribonuclease	*XRN2*	Spermatogenesis
G3RRF4	Alpha fetoprotein		Ovulation from ovarian follicle, progesterone metabolic process
F7F3Q9	Fructose-bisphosphate aldolase		Binding of sperm to zona pellucida

#### Network interaction of identified extraembryonic membrane proteins with reproductive process information

**Fig. 8:**
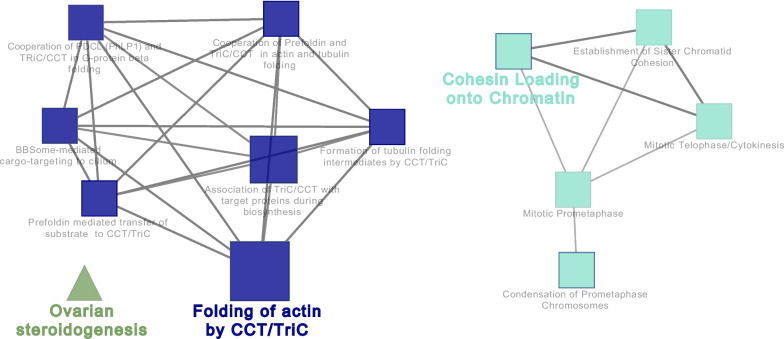
Visualization of the functional analysis of the proteins involved in the reproductive process annotation in extraembryonic membranes of pregnant sows at d 18 and 24 of gestation. The size of the nodes indicates the pathway´s enrichment significance. The following ClueGO parameters were used: *P*-value cut-off = 0.001; Statistical test used the enrichment/depletion (two-sided hypergeometric test); Bonferroni step down with minimum GO level 2; maximum GO level 6; kappa score threshold equal to 0.4. GO = Gene Ontology. Network includes the interaction of RAD21, SMC1A, SMC3, CCT3, CCT4, CCT8 and TCP1 proteins

**Fig. 9:**
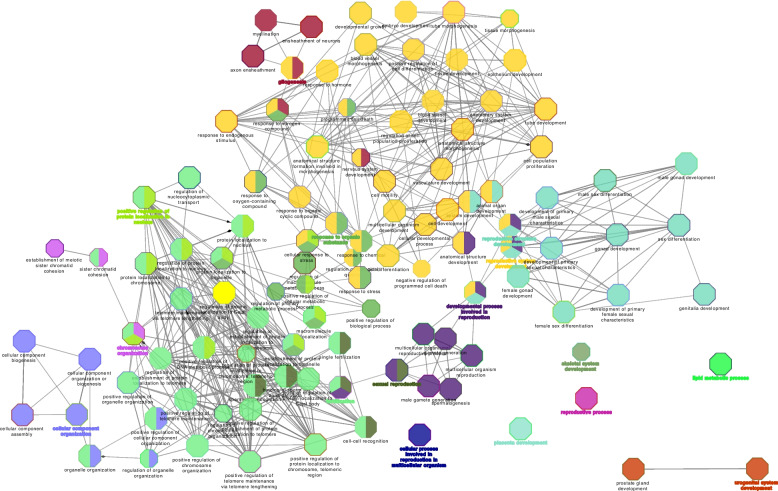
Visualization of the biological gene ontology analysis of the proteins involved in the reproductive process annotation in extraembryonic membranes of pregnant sows at d 18 and 24 of gestation. The size of the nodes indicates the pathway´s enrichment significance. The following ClueGO parameters were used: *P*-value cut-off = 0.001; Statistical test used the enrichment/depletion (two-sided hypergeometric test); Bonferroni step down with minimum GO level 2; maximum GO level 6; kappa score threshold equal to 0.4. GO = Gene Ontology. Network includes the interaction of CCDC134, HSD17B2, HSP90AB1, KRT19, MAPK14, NOTCH2, PRDX3, PTGS2, RTCB, ST14, ACE, APOB, BAX, CCDC134, CDK1, CELF1, CTNNB1, DHX36, DLG1, HSD17B2, ITGB4, KDR, KRT19, LRP2, MSH2, NOTCH1, NOTCH2, PAFAH1B1, PDGFRA, PRKACA, RAD23B, RTCB, SEPTIN7, SMAD1 and ST14 proteins

Cytoscape ClueGO analysis of the 94 proteins with reproductive process information revealed the interaction of proteins in various REACTOME pathways, such as the interaction of RAD21 (RAD21 cohesin complex component), SMC1A (structural maintenance of chromosomes) and SMC3 (structural maintenance of chromosomes) proteins network in cohesin loading onto chromatin and of CCT3 (T-complex protein 1 subunit gamma), CCT4 (T-complex protein 1 subunit delta), CCT8 (T-complex protein 1 subunit theta), TCP1 (T-complex protein 1 subunit alpha) proteins network in folding of actin by CCT/TriC pathways (Fig. 8, Table S14). ClueGO analysis also revealed the interaction of a large number of extraembryonic membrane proteins in various GO terms of biological process (Fig. 9, Table S15), such as placenta development (CCDC134 (coiled-coil domain containing 134), HSD17B2 (estradiol 17-beta dehydrogenase 2), HSP90AB1 (heat shock protein 90 alpha family class B member 1), KRT19 (keratin 19), MAPK14 (mitogen-activated protein kinase 14), NOTCH2 (notch receptor 2), PRDX3 (peroxiredoxin 3), PTGS2 (prostaglandin G/H synthase 2), RTCB (RNA-splicing ligase RtcB homolog), ST14 (ST14 transmembrane serine protease matriptase)) and embryo development (ACE (angiotensin-converting enzyme), APOB (apolipoprotein B), BAX (apoptosis regulator Bax), CCDC134 (coiled-coil domain containing 134), CDK1 (cell division cycle 2), CELF1 (CUGBP Elav-like family member 1), CTNNB1 (catenin beta 1), DHX36 (RNA helicase), DLG1 (discs large MAGUK scaffold protein 1), HSD17B2, ITGB4 (integrin beta), KDR (receptor protein-tyrosine kinase), KRT19 (keratin, type I cytoskeletal 19 isoform X1), LRP2 (low-density lipoprotein receptor-related protein), MSH2 (DNA mismatch repair protein), NOTCH1 (neurogenic locus notch homolog protein 1), NOTCH2 (notch receptor 2), PAFAH1B1 (platelet-activating factor acetylhydrolase IB subunit alpha), PDGFRA (platelet-derived growth factor receptor alpha), PRKACA (cAMP-dependent protein kinase), RAD23B (UV excision repair protein RAD23), RTCB (RNA-splicing ligase RtcB homolog), SEPTIN7 (septin), SMAD1 (mothers against decapentaplegic homolog), and ST14 (ST14 transmembrane serine protease matriptase)).

### Validation study

**Fig. 10:**
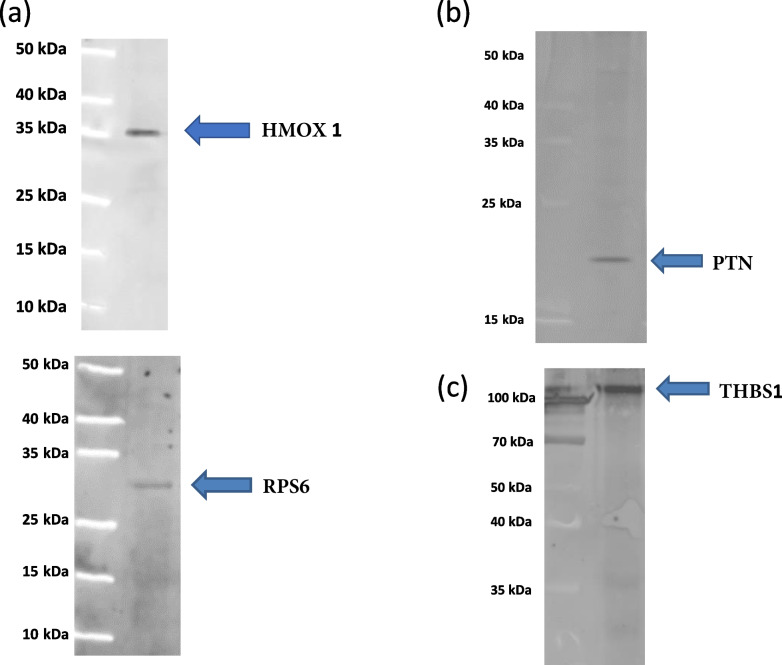
Representative validation by Western blot of HMOX1 (hemo oxidase 1) and RPS6 (40S ribosomal protein) proteins in the porcine endometrium of pregnant sows (**a**) of PTN protein (pleiotrophin) in the porcine endometrium of non-pregnant sows (**b**) and THBS1 protein (thrombospondin1) in the extraembryonic membranes of pregnant sows (**c**)

To validate the results of our qualitative LC-MS/MS proteomic study, 4 of the identified proteins were selected for Western blot analysis (Fig. 10). The selected proteins were HMOX1 (heme oxygenase 1) and RPS6 (40S ribosomal protein) proteins from the endometrium of PS, PTN (pleiotrophin) protein from the endometrium of NPS and THBS1 (thrombospondin 1) protein from the extraembryonic membranes. This selection was based on the availability of the antibodies for the study of porcine proteins and on the important role of HMOX1 and RPS6 in the development of endometrial receptivity in the case of PS proteins. The Western blot validation experiment confirmed the presence of HMOX1 and RPS6 proteins in the endometrium of PS and PTN protein in the endometrium of NPS as well as the presence of THBS1 protein in the extraembryonic membranes, supporting the proteomic data.

## Discussion

This study presents a comprehensive characterization of the endometrial proteome during d 18 and 24 of both PS and NPS, with the major objective of creating a catalog of proteins that are present during this crucial phase of porcine gestation. This period is important as it occurs immediately after trophoblast attachment and marks evidence of early pregnancy. This study is the first of its kind to perform such a large-scale characterization. We also analyzed the proteomic profile of extraembryonic membranes to better understand the functionality of the placenta at this critical stage of pregnancy.

Proteomics is a highly dynamic element that changes in an organism, tissue, or cell because of environmental changes, stress, or a physiological or pathological condition. It provides valuable information that cannot be derived from genomic or transcriptomic studies [19]. Quantitative analysis of proteins is essential to obtain relevant information on specific biological system; however, to obtain an overall view of the proteomic profile, qualitative proteomic analysis is also required. This qualitative information could contribute to a better understanding of the local maternal response during implantation and provide possible clues to reduce pregnancy failure. For our proteomic study, we used LC-MS/MS operated in data-dependent acquisition mode and it supported the detection of 3,254, 3,457 and 3,968 proteins at high-confidence in PS and NPS endometria and membranes, respectively.

Previous reports have identified endometrial proteins during various pregnancy time points using different proteomic techniques. The most classically used has been the two-dimensional (2D) gel electrophoresis followed by mass spectrometry (MS) or MALDI TOF-TOF analysis to identify protein spots. The 2D-MS/MALDI TOF-TOF has been used to identify 70 to 500 proteins in the peri-implantation period (d 9 to 16) and 820 proteins at mid-pregnancy (d 40 to 93) in the endometrium of pregnant and non-pregnant sows [11–13, 20, 21]. These proteomic studies have provided information on the dynamic physiological protein profile and how it may relate to maternal recognition and pregnancy. In recent years, advances in proteomics, particularly technologies based on MS, have allowed us to increase sensitivity, confidence and proteomic coverage compared to those of traditional methods. Using LC-MS/MS, we were able to identify a substantial number of proteins in our study. These findings are consistent with the 3,185 proteins that were previously identified by Wang et al. [14] in endometrial tissue during mid-pregnancy.

When we explored the endometrium proteome of PS and NPS, we first observed that the 10 high abundant proteins found in the two proteomes were proteins involved in immune system process and proteins with binding and cytoskeletal organization functions, both of which are involved in the structural support of the endometrium. In general, the functions of these 10 proteins are consistent with the basic development of the endometrium, which is constantly remodeling. To further classify the identified proteins in the endometrial tissues, we conducted an evaluation of their functional gene ontology in each endometrial proteome. As expected, both proteomes exhibited a similar pattern of functional categorization. For this reason, and to identify exclusive proteins during these critical days of pregnancy, we created a Venn diagram by comparing all proteins identified in the endometrium of PS with all proteins identified in the NPS endometrium during the corresponding days of the cycle. To the best of our knowledge, this study provides the largest number of unique proteins identified in the endometrium of PS and NPS during the peri-implantation period (1,501 and 1,704 proteins, respectively). The proteins identified in these two lists may contribute to a better understanding of the biology of the endometrium during the implantation phase and serve as valuable tools for identifying the main regulators responsible for endometrial remodeling and the appropriate development of pregnancy. To gain a more extensive insight into the biological networks of these distinctive proteins, we utilized ClueGO, which is a Cytoscape plug-in that greatly enhances the biological interpretation of genes and proteins by selecting relevant GO terms and pathways and displaying them in well-organized networks.

The unique proteins involved in the immune system of PS are of particular interest, given that the modulation of the endometrial immune system is crucial for endometrial function and implantation [9, 22]. Among the 53 PS-unique proteins annotated, 10 proteins were involved in cytokine signaling in immune system pathway in ClueGo analysis. The immunological status of the female reproductive tract is dynamic and subject to changes depending on both the estrous cycle and gestational stage. Research has shown that the mid-implantation stage (d 18) is characterized by an increase in pro-inflammatory cytokine expression, which facilitates embryo implantation interactions between the placenta and uterus during porcine pregnancy [23, 24]. Conversely, during the late implantation phase (d 20–28), anti-inflammatory expression is observed to support placental and conceptus development [22]. Practically all identified PS-unique proteins involved in immune system process were pro-inflammatory proteins, with the exception of HMOX1, which has anti-inflammatory properties. Based on this, it is possible that a pro-inflammatory environment predominates from d 18 to d 24 of gestation, with a rapid shift towards an anti-inflammatory environment by d 25. Furthermore, we also identified that several PS-unique proteins of PS endometrium were involved in the Toll Like Receptor 4 (TLR4) cascade pathway. Toll-like receptors are important sensors of the innate immune system that promote the production of important pro-inflammatory molecules such as cytokines and chemokines [25]. Activation of TLR4 during pregnancy has been shown to bridge innate and adaptive immunity to protect the developing fetus from pathogens [26]. Among the identified PS-unique proteins involved in the TLR4 cascade, LBP and CD14, are pattern recognition receptors or proteins capable of recognizing molecules commonly found in pathogens and acting as the first line of defense [27]. Additionally, MAPK14 and MAP2K1 proteins, play an important role in the cascades of cellular responses that lead to the direct activation of transcription factors implicated in inflammatory responses [28]. A healthy pregnancy requires a balance between immunological defense and immunosuppression to ensure semi allogenic fetal development. The involvement of the PS endometrial proteins identified in our study in the TLR4 cascade may indicate that an active maternal immune system is crucial to combat the upcoming intrauterine microorganism challenge. Herein, we have identified several proteins that may contribute to the modulation of the endometrial immune system, at least between d 18 and 24 of gestation.

The implantation and maintenance of pregnancy is closely related to the restructuring of the cytoskeleton and the remodeling of adhesive contacts with the extracellular matrix (ECM) [29]. Based on their biological adhesion annotation, all PS- and NPS-unique proteins of the endometrium are proteins with different structural functions that control cell behavior such as proliferation, adhesion, and migration and regulate cell differentiation and death. The analysis by Cytoscape ClueGO of the NPS-unique proteins showed that most of the proteins with biological adhesion annotation were involved in focal adhesion, ECM-receptor interaction, PI3K-Akt signaling and regulation of actin cytoskeleton pathways. The laminin (LAMA3, LAMA5) and integrin (ITGA9, ITGAX, ITGB4, ITGA3) proteins, as well as FN1 and other NPS-unique proteins involved in these pathways, are implicated in cyclic remodeling of the endometrial epithelium in the absence of embryo implantation. Regarding the PS-unique proteins identified in the biological adhesion category of the present study, these proteins are associated not only with the organization of the cytoskeleton but also with the adhesion process. Of the 23 PS-unique endometrial proteins, Cytoscape ClueGO analysis revealed that EMILIN1, HAPLN1, TNC, and ITGAM are involved in the network of ECM organization. EMILIN1 protein plays a role in creating elastic fibers and promoting cell adhesion. This protein has been identified as a ligand molecule in the endometrium that interacts with receptors found on bovine d 16 conceptuses [30]. The protein TNC is involved in embryogenesis and plays an important role in endometrial proliferation [31]. It has been established that the integrin gene *ITGAM* also plays a crucial role during implantation, as the injection of an ITGAM antibody into the uterine lumen of early pregnant mice results in pregnancy loss [32]. The expression of this gene, which is related to cell adhesion processes, was found to be upregulated in the luminal epithelium of the pig endometria collected at d 14 of pregnancy [33]. HAPLN1 is another well-known ECM protein that plays a role in the adhesion process and is also expressed in various tissues such as the intestine and human placenta [34]. In addition to the above proteins, in the present study we identified other unique proteins in PS endometrium that may be important for the adhesion of embryos to the surface of the endometrium. One of these proteins is the TGFB1. Among other processes, TGFB1 has an important function in angiogenesis, embryogenesis and trophoblast attachment [35] and its activation at d 15–16 of pregnancy in pigs was suggested by Kolakowska et al. [20]. We confirm the presence of TGFB1 in the endometrium of PS at d 18–24 of gestation, which may indicate its importance in the proper course of pregnancy. In addition, the detection of high expression levels of HAPLN1 and TGFB1 in the endometrium of pregnant sows at mid-pregnancy [14] indicates that these proteins could play a crucial role in both implantation and the maintenance of pregnancy. The PS endometrial proteins with biological adhesion annotation identified in the present study, could be candidate proteins potentially involved in embryo-maternal dialogue.

The proteins with reproductive process annotation are particularly important for the interpretation of the PS proteome. Among these, we have identified proteins that are involved in and contribute to the successful establishment of pregnancy. Proteins such as RPS6, HECTD1, HSD172B2, MAP2K1 and MAPK14 are involved in ClueGO analysis in the reproductive structure and system development, and placenta development GOs, and could be responsible for the development of endometrial receptivity. It has been reported that RPS6 is a downstream target protein of the phosphatidylinositol-3-kinasa-AKT (PI3K/AKT) pathway. This signaling system plays an important function during the implantation phase of porcine pregnancy by stimulating the migration and attachment of trophectoderm and luminal epithelial cells [36]. HECTD1, identified here in PS endometrial tissue, has been previously described as indispensable for normal embryogenesis and fetal survival in mice [37] and as an important protein for human [38] and mouse [39] placentation. Therefore, it is reasonable to assume that this protein may be involved in the development of the junctional zone between the endometrium and extraembryonic membranes in pigs. High expression of *HSD17B2* has been observed in the horse endometrium [40]. This molecule exerts a local antiestrogenic effect by converting active estradiol to inactive estrone in endometrial epithelial cells [41]. MAPK14, which is involved in mitogen-activated protein kinase pathways, mediates embryonic responses and plays an important role in the growth of human embryos [42]. Similarly, MAP2K1 is important for the full expansion of the fetal-maternal exchange area and its loss of function leads to embryonic death due to placental defects [43]. In addition, we have identified other PS-unique proteins involved in the development of pregnancy, such as HPGD and DLG1. It has been reported that the expression and activity of HPGD is regulated by the progesterone and that this molecule may be involved in the protection of pregnancy from prostaglandin at the beginning of pregnancy [44]. The involvement of DLG1 protein in pregnancy events is completely unknown, but it has been shown to be involved in dynamic tissue movement [45] and its loss of function leads to complete neonatal lethality [46].

In the pig, as in other mammals, the establishment of reciprocal interactions between the embryo-fetus and its associated extraembryonic membranes, particularly the chorio(allantois) and the endometrium is necessary for the success of implantation and the maintenance of a healthy pregnancy. The placenta in the pig is not invasive. As a type of epitheliochorial placenta, around d 12–13 of gestation, the adhesion of embryos to the uterine epithelium starts [47, 48]. In the present study, we identified for the first time the proteome of extraembryonic membranes of PS at early stage of pregnancy (d 18 and 24). This study is primary descriptive and these proteins can be a good starting point for the discovery of proteins related to placental development and the implantation process. As expected, the functions of the high abundance proteins in the extraembryonic membranes were related to trophoblastic cell proliferation and placental tissue organization [49], as well as vascularization and angiogenesis, which are necessary for utero-placental blood flow to supply nutrients to the fetus [50]. Out of the 3,968 proteins identified, we were interested in the 94 proteins annotated with reproductive processes. Our goal was to identify and characterize the protein-protein interaction networks that govern placental function. In this study we were particularly interested in the interaction among proteins related placenta and embryo development terms. Among them, HSD17B2, MAPK14, ITGB4, and DLG1 could be related to the balanced coordination of signaling pathways between membranes, endometrium, and embryo-fetus because they are also expressed in the endometrium of PS. The current network results could be important for identifying significant proteins as candidates for future functional studies of the placenta. Of potential interest is the identification of the protein NOTCH2. During early gestation, the vascular development of the fetus and extraembryonic tissues has been found to be significantly impacted by Notch signaling molecules. For instance, the smaller size of cloned porcine extraembryonic tissues at d 26 of gestation, which ultimately leads to embryonic losses in the first trimester, has been attributed to the low expression of these molecules [51].

## Conclusion

In this study, we have detected proteins that can play an important role in the development of the endometrium and extraembryonic membranes during embryo implantation (d 18 and 24 of gestation) in pigs. The identification of these proteins may help to clarify the crosstalk between the endometrium, conceptus and membranes at that critical period of pregnancy.

### Supplementary Information

**Additional file 1:** **Table S1.** Complete list of the 3,254 proteins identified in endometrium of pregnant sows during implantation (d 18 and 24 of gestation) validated with a peptide confidence threshold of 95% and a false discovery rate (FDR) of 1%.** Table S2.** Complete list of the 3,457 proteins identified in endometrium of non-pregnant sows at d 18 and 24 of cycle validated with a peptide confidence threshold of 95% and a false discovery rate (FDR) of 1%.** Table S3.** Complete list of the 3,968 proteins identified in extra-embryonic membranes of pregnant sows at d 18 and 24 of gestation validated with a peptide confidence threshold of 95% and a false discovery rate (FDR) of 1%. **Table S4.** Complete list of 1,751 unique proteins in endometrium of pregnant sows. **Table S5.** Complete list of 1,704 unique proteins in endometrium of non-pregnant sows.** Table S6.** Complete list of 1,753 common proteins in endometrium of pregnant and non-pregnant sows. **Table S7.** Functional group analysis with their associated proteins in unique pregnant sow’s proteins with immune system annotation (data generated by ClueGO plugin).** Table S8.** Functional group analysis with their associated proteins in unique pregnant sow’s proteins with biological adhesion annotation (data generated by ClueGO plugin).** Table S9.** Functional group analysis with their associated proteins in unique pregnant sow’s proteins with reproductive process annotation (data generated by ClueGO plugin).** Table S10.** The Gene Ontology (GO) Biological functional groups analysis with their associated proteins in unique pregnant sow’s proteins with reproductive process annotation (data generated by ClueGO plugin).** Table S11.** Functional group analysis with their associated proteins in unique non-pregnant sow’s proteins with immune system annotation (data generated by ClueGO plugin).** Table S12.** Functional group analysis with their associated proteins in unique pregnant sow’s proteins with biological adhesion annotation (data generated by ClueGO plugin).** Table S13. **The Gene Ontology (GO) Biological functional groups analysis with their associated proteins in unique pregnant sow’s proteins with reproductive process annotation (data generated by ClueGO plugin). **Table S14.** Functional group analysis with their associated proteins in pregnant sow’s extraembryonic membrane proteins with reproductive process annotation (data generated by ClueGO plugin). **Table S15.** The Gene Ontology (GO) Biological functional groups analysis with their associated proteins in pregnant sow’s extraembryonic membrane proteins with reproductive process annotation (data generated by ClueGO plugin).

## Data Availability

All data generated or analyzed during this study are included in this published article [and its supplementary information files]. Further inquiries can be directed to the corresponding author. The mass spectrometry proteomics data have been deposited to the ProteomeXchange Consortium via the PRIDE [17] partner repository with the dataset identifier PXD042565.

## Abbreviations

ACN: Acetonitrile
1D SDS-PAGE: One-dimensional sodium dodecyl sulfate-polyacrylamide gel electrophoresis
2D: Two-dimensional
ECM: Extracellular matrix
FA: Formic acid
FDR: False discovery rate
GO: Gene Ontology
LC-MS: Liquid chromatography-mass spectrometry
MS: Mass spectrometry
NPS: Non-pregnant sows
PS: Pregnant sows
TFA: Trifluoroacetic acid
TOF: Time of flight
